# The empathic brain and its dysfunction in psychiatric populations: implications for intervention across different clinical conditions

**DOI:** 10.1186/1751-0759-1-22

**Published:** 2007-11-16

**Authors:** Jean Decety, Yoshiya Moriguchi

**Affiliations:** 1 Departments of Psychology and Psychiatry, The University of Chicago, 5848 S. University Avenue, Chicago, IL 60637, USA; 2 Department of Psychosomatic Research, National Institute of Mental Health, National Center of Neurology and Psychiatry, Ogawa-Higashi Cho, Kodaira City, Tokyo, 187-8551, Japan

## Abstract

Empathy is a concept central to psychiatry, psychotherapy and clinical psychology. The construct of empathy involves not only the affective experience of the other person's actual or inferred emotional state but also some minimal recognition and understanding of another's emotional state. It is proposed, in the light of multiple levels of analysis including social psychology, cognitive neuroscience and clinical neuropsychology, a model of empathy that involves both bottom-up and top-down information processing underpinned by parallel and distributed computational mechanisms. The predictive validity of this model is explored with reference to clinical conditions. As many psychiatric conditions are associated with deficits or even lack of empathy, we discuss a limited number of these disorders including psychopathy/antisocial personality disorders, borderline and narcissistic personality disorders, autistic spectrum disorders, and alexithymia. We argue that future clinical investigations of empathy disorders can only be informative if behavioral, dispositional and biological factors are combined.

## Introduction

There is no dispute that empathy – the capacity to share and understand emotional states of others in reference to oneself – plays a critical role in human interpersonal engagement and social interaction. In recent decades there has been a flurry of interest in the domain of empathy in various academic domains including philosophy of mind, social psychology, developmental science and cognitive neuroscience. Moreover, genuine cross-disciplinary work has begun to investigate the cause, correlates and consequences of empathy in typically developing children and healthy individuals.

We believe that it is time to bring together conceptual theories of empathy and recent empirical data in the context of psychopathology. Two reasons motivated us to address empathy in such a context. First, much of psychotherapy depends on helping people to analyze and understand their inner world to enable them to relate more appropriately to their conspecifics. Second, many psychiatric disorders are associated with empathy-related deficits. For instance it is well documented that antisocial individuals lack concern for others. The DSM-IV identifies a deficiency in empathy as one of the essential features of narcissistic personality disorder. Empathic deficits constitute a primary source of the autistic syndrome. This list, far from exhaustive, illustrates the complexity of the psychological construct of empathy, which explains why such a variety and heterogeneity of mental disorders can be associated with empathy.

The goal of this paper is to provide an overarching framework of empathy that articulates different components with recent empirical data from neural science (including neuropsychology). After clearing up definitional issues, we present our framework, which considers that empathy relies on both bottom-up and top-down information processing and involves parallel and distributed processing in a number of dissociable computational mechanisms. Shared neural representations, self-other awareness, mental flexibility and emotion regulation constitute the basic macro-components of empathy, which are mediated by specific and interacting neural systems. Consequently, empathy-related disorders may occur from dysfunction or disruption on each of these components. We then discuss several psychopathological conditions, which share lack of empathy and demonstrate how they can be related to this framework.

## What do we mean by empathy

There is an overabundance of operational definitions of empathy. The construct of empathy denotes, at a phenomenological level of description, a sense of similarity between the feelings one experiences and those expressed by others [1]. It can be conceived of as an interaction between any two individuals, with one experiencing and sharing the feeling of the other. Yet, empathic is not a clear-cut expression. Specifically, empathy poses a paradox, as sharing of feelings does not necessarily imply that one will act or even feel impelled to act in a supportive or sympathetic way (empathy's paradox is that this ability may be used for both helpful and hurtful purposes). Empathy is a source of altruistic motivation, which under certain circumstances may produce behavior that might be judged moral but under other circumstances may produce behavior that might be judged immoral [2]. Moreover, the social and emotional situations eliciting empathy can become quite complex depending on the feelings experienced by the observed and the relationship of the target to the observer [3]. The caveats demonstrate that understanding others and experiencing their feelings manifests in relation to oneself, illustrating the social nature of the self, its inherent intersubjectivity.

Humans are indeed an intrinsically social and gregarious species. And virtually all of their actions (including their thoughts, desires, and feelings) are directed toward or are produced in response to others [4]. Our survival critically depends on social interactions with others. However, we are also reasoning beings with flexibility and reflexivity. This does not mean that basic emotion processing does not play a role. Rather, emotions are embedded in reasoning involving evaluative appraisals.

Here we view empathy as a multidimensional construct to account for the sense of sharing and understanding the subjective experience of others. Thus empathy includes aspects of emotion communication, self-awareness and theory of mind.

## The evolution of empathy

Natural selection has fine-tuned the mechanisms that serve the specific demands of each species' ecology, and social behaviors are best understood in the context of evolution. However, one needs to point out that the literature in comparative psychology and ethology is plagued by the same limitations as the human literature regarding the difficulty to clearly differentiate true empathic reactions from other emotional reactions such as personal distress.

The phylogenic origin of behaviors associated with social engagement has been linked to the evolution of the autonomic nervous system and how it relates to emotion. According to Porges[5], social approach or withdraw stem from the implicit computation of feelings of safety, discomfort, or potential danger. He proposed that the evolution of the autonomic nervous system (sympathetic and parasympathetic systems) provides a means to understand the adaptative significance of the mammalian affective processes including empathy and the establishment of lasting social bonds. These basic evaluative systems are associated with motor responses that aid the adaptive responding of the organism. At this primitive level, appetitive and aversive behavioral responses are modulated by specific neural circuits in the brain that share common neuroarchitecture among mammals [6]. These brain systems are genetically hard-wired to enable animals to respond unconditionally to threatening, or appetitive, stimuli using specific response patterns that are most adaptive to the particular species and environmental condition. The limbic system, which includes the hypothalamus, the parahippocampal cortex, the amygdala, and several interconnected areas (septum, basal ganglia, nucleus accumbens, insula, retrospenial cingulate cortex and prefrontal cortex) is primarily responsible for emotion processing. What unite these regions are their roles in motivation and emotion, mediated by connections with the autonomic system. The limbic system also projects to the cingulate and orbitofrontal cortices, which are involved with the regulation of emotion.

There is evidence for a lateralization of emotion processing in humans and primates, which has been marshaled under two distinct theories. One theory states that the right hemisphere is primarily responsible for emotional processing [7], while another one suggests that the right hemisphere regulates negative emotion and the left hemisphere regulates positive emotion [8]. This asymmetry is anatomically based on an asymmetrical representation of homeostatic activity that originates from asymmetries in the peripheral autonomic nervous system, and fits well with the homeostatic model of emotional awareness, which posits that emotions are organized according to the fundamental principle of autonomic opponency for the management of physical and mental energy [9]. Supporting evidence for the lateralization of emotion comes from neuroimaging studies and neuropsychological observations with brain damaged patients, but also studies in non-human primates. In one study, tympanic membrane temperature (Tty) was used to assess asymmetries in the perception of emotional stimuli in chimpanzees [10]. The tympanic membrane is an indirect, but reliable, site from which to measure brain temperature, and is strongly influenced by autonomic and behavioral activity. In that study, chimpanzees were shown positive, neutral, and negative emotional videos depicting scenes of play, scenery, and severe aggression, respectively. During the negative emotion condition, right Tty was significantly higher than the baseline temperature. This effect was relatively stable, long lasting, and consistent across individuals. Temperatures did not change significantly from baseline in the neutral or positive emotion condition, although a significant number of measurements showed increased left Tty during the neutral emotion condition. These data suggest that viewing emotional stimuli results in asymmetrical changes in brain temperature, in particular increased right Tty during the negative emotion condition, evidence of emotional arousal in chimpanzees, and in providing support right hemispheric asymmetry in our closest living ancestor.

At the behavioral level it is evident from the descriptions of comparative psychologists and ethologists that behaviors homologous to empathy can be observed in other mammalian species. Notably, a variety of reports on ape empathic reactions suggests that, apart from emotional connectedness, apes have an explicit appreciation of the other's situation [11]. A good example is consolation, defined as reassurance behavior by an uninvolved bystander towards one of the combatants in a previous aggressive incident [12]. De Waal [11] has convincingly argued that empathy is not an all-or-nothing phenomenon, and many forms of empathy exist between the extremes of mere agitation at the distress of another and full understanding of their predicament. Many other comparative psychologists however view empathy as a kind of induction process by which emotions, both positive and negative, are shared, and by which the probabilities of similar behavior are increased in the participants. In the view developed in this paper, this is a necessary but not a sufficient mechanism to account for the full-blown ability of human empathy. However it does provide the basic primitive, yet crucial mechanism on which empathy develops. Indeed, some aspects of empathy are present in other species, such as motor mimicry and emotion contagion (see [13]). For instance, Parr [14] conducted an experiment in which peripheral skin temperature (indicating greater negative arousal) was measured in chimpanzees while they were exposed to emotionally negative video scenes. Results demonstrate that skin temperature decreased when subjects viewed videos of conspecifics injected with needles or videos of needles themselves, but not videos of a conspecific chasing the veterinarian. Thus, when chimpanzee are exposed to meaningful emotional stimuli, they are subject to physiological changes similar to those observed during fear in humans, which is similar to the dispositional effects of emotional contagion [15].

In humans, the construct of empathy accounts for a more complex psychological state than the one associated with the automatic sharing of emotions. Like in other species, emotions and feelings may be shared between individuals, but humans are also able to intentionally "feel for" and act on behalf of other people whose experiences may differ greatly from their own [16,17]. This phenomenon, called empathic concern or sympathy, is often – but not always – associated with prosocial behaviors such as helping kin, and has been considered as a chief enabling process for altruism [17]. Notably, Wilson [18] suggested that empathic helping behavior has evolved because of its contribution to genetic fitness (kin selection). In humans and other mammals, an impulse to care for offspring is almost certainly genetically hard-wired. It is far less clear that an impulse to care for siblings, more remote kin, and similar non-kin is genetically hard-wired [19]. The emergence of altruism, of empathizing with and caring for those who are not kin is thus not easily explained within the framework of neo-Darwinian theories of natural selection. Social learning explanations of kinship patterns in human helping behavior are thus highly plausible. However, one of the most striking aspects of human empathy is that it can be felt for virtually any target – even targets of a different species. In addition, as emphasized by Harris [20], humans, unlike other primates, can put their emotions into words, allowing them not only to express emotion but also to report on current as well as past emotions. These reports provide an opportunity to share, explain, and regulate emotional experience with others that is not found in other species. Conversation helps to develop empathy, for it is often here that one learns of shared experiences and feelings. Moreover, this self-reflexive capability (which includes emotion regulation) may be an important difference between humans and other animals [21].

Interestingly two key regions, the anterior insula and anterior cingulate cortex (ACC), involved in affective processing in general and empathy in particular have singularly evolved in apes and humans. Cytoarchitectonic work by Allman and colleagues [22] indicates that a population of large spindle neurons is uniquely found in the anterior insula and anterior cingulate of humanoid primates. Most notably, they reported a trenchant phylogenetic correlation, in that spindle cells are most numerous in aged humans, but progressively less numerous in children, gorillas, bonobos and chimpanzees, and nonexistent in macaque monkeys. Craig [23] recently suggested that these spindle neurons interconnect the most advanced portions of limbic sensory (anterior insula) and limbic motor (ACC) cortices, both ipsilaterally and contralaterally, which, in sharp contrast to the tightly interconnected and contiguous sensorimotor cortices, are situated physically far apart as a consequence of the pattern of evolutionary development of limbic cortices. Thus, the spindle neurons could enable fast, complex and highly integrated emotional behaviors. In support of this view, convergent functional imaging findings reveal that the anterior insula and the anterior cingulate cortices are conjointly activated during all human emotions. This, according to Craig [24], indicates that the limbic sensory representation of subjective "feelings" (in the anterior insula) and the limbic motor representation of volitional agency (in the anterior cingulate) together form the fundamental neuroanatomical basis for all human emotions, consistent with the definition of an emotion in humans as both a feeling and a motivation with concomitant autonomic sequelae [25].

Overall, this evolutionary conceptual view is compatible with the hypothesis that advanced levels of social cognition may have arisen as an emergent property of powerful executive functioning assisted by the representational properties of language [26]. However, these higher levels operate on previous levels of organization, and should not be seen as independent of, or conflicting with one another. Evolution has constructed layers of increasing complexity, from non-representational (e.g., emotion contagion) to representational and meta-representational mechanisms (e.g., sympathy), which need to be taken into account for a full understanding of human empathy.

## The components of empathy

For many psychologists, empathy implies at least three different processes: feeling what another person is feeling; knowing what another person is feeling; and having the intention to respond compassionately to another person's distress [1]. Yet, regardless of the particular terminology that is used, there is broad agreement among scholars on three primary aspects: 1) an affective response to another person, which often, but not always, entails sharing that person's emotional state, 2) a cognitive capacity to take the perspective of the other person, and 3) some self-regulatory and monitoring mechanisms that modulate inner states (e.g., [16,17,27,28]). According to Ickes [29], empathy is a complex form of psychological inference in which observation, memory, knowledge, and reasoning are combined to yield insights into the thoughts and feelings of others. As such, empathy involves not only the affective experience of the other person's actual or inferred emotional state but also some minimal recognition and understanding of another's emotional state (or most likely emotional state). This latter definition captures the multidimensional nature of empathy and makes explicit reference to some minimal mentalizing capacity. This latter concept refers to the broad social-cognitive ability used by humans to explain and predict their own behavior and that of others by attributing to them independent mental states, such as belief, desires, emotions or intentions [30].

The model proposed here suggests that four major functional components dynamically interact to produce the experience of empathy:

**1**. Affective sharing between the self and the other, based on the automatic perception-action coupling and resulting shared representations.

**2**. Self-awareness. Even when there is some temporary identification between the observer and its target, there is no confusion between self and other.

**3**. Mental flexibility to adopt the subjective perspective of the other.

**4**. Regulatory processes that modulate the subjective feelings associated with emotion.

**Figure 1:**
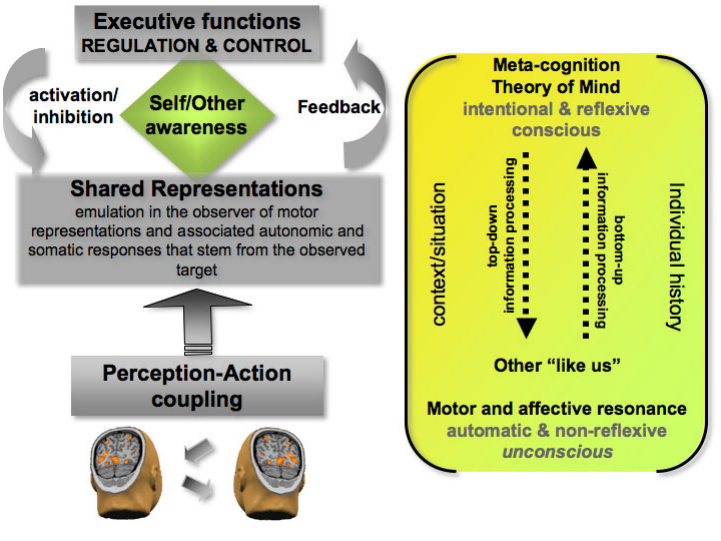
Schematic representation of the bottom-up (i.e., direct matching between perception and action), and top-down (i.e., regulation and control) information processes involved in empathy. These two levels of processing are interrelated. The lower level, which is automatically activated (unless inhibited) by perceptual input, accounts for emotion sharing which leads to the implicit recognition that others are like us. Executive functions, implemented in the prefrontal cortex, serve to regulate both cognition and emotion, notably through selective attention and self-regulation. This meta-level is continuously updated by bottom-up information, and in return controls the lower level by providing top-down input. Thus, top-down regulation, through executive functions, modulates low levels and adds flexibility, making the individual less dependent on external cues. The meta-cognitive feedback plays a crucial role in taking into account one's own mental competence in order to react (or not) to the affective states of others. However, subcortical systems do not lose their basic function, they may give up some of their autonomy in terms of the degree to which higher cortical systems can modulate their functions or regulate the emotional experience (adapted from [168–170]).

In this view, none of these components can account solely for the potential of human empathy. The four components are intertwined and interact with one another to produce the subjective experience of human empathy. For instance, sharing emotion without self-awareness corresponds to the phenomenon of emotional contagion, which takes the form of 'total identification without discrimination between one's feelings and those of the other [11]. This model of empathy combines both representational aspects, i.e., memories that are localized in distributed neural networks that encode information and, when temporarily activated, enable access to this stored information, and processes, i.e., computational procedures that are localized and are independent of the nature or modality of the stimulus that is being processed (see Figure 1).

Like many emotion-related processes, some components involved in empathy occur implicitly, without awareness, in a bottom-up fashion. This is the case with the emotion-sharing and motor mimicry aspects. Other components require explicit top-down processing, such as perspective-taking, representing our own thoughts and feelings as well as those of others, and also some aspects of emotion regulation.

## Affective sharing between self and other

In primates, the ability to understand emotional states of others is critical for maintaining social interactions. One powerful way to learn about the emotions expressed by others is through emotional contagion. More complex forms of emotions (such as social emotions) require the awareness of one's own feelings in relation with, or in response to social interaction.

Emotional expression and perception are an integral part of any social interaction [31]. Bodily expressions constitute an external, perceivable indication of people's intentions and emotions. At one level, emotional expressions are governed by rules and can be elicited by simple stimuli, as in the example of disgust in the presence of bitter taste. However, humans as well as other animals also use bodily expressions to communicate various types of information to members of their own species. Understanding other people's emotional signals has clear adaptive advantages and is especially important in the formation and maintenance of social relationships.

Social psychological research shows that humans mimic unintentionally and unconsciously a wide range of behaviors, such as accents, tone of voice, rate of speech, posture and mannerisms, as well as moods (e.g., [32]). This tendency to automatically mimic and synchronize one's own emotional behavior with that of others, also known as the phenomenon of emotion contagion, facilitate the smoothness of social interaction and may even foster empathy [33]. For instance, a study demonstrated that participants who had been mimicked by the experimenter were more helpful and generous toward other people than non-mimicked participants [34]. That study also found that these beneficial consequences of mimicry were not restricted to behavior directed toward the mimicker, but included behavior directed toward people not directly involved in the mimicry situation. Interestingly, individuals with autism, who are profoundly impaired in social and emotional abilities, do not show spontaneous mimicry, but they perform voluntary mimicry well [35]. Such a core deficit in involuntary motor resonance may be the seed for their profound impairment in basic emotional connectedness.

This automatic mapping between self and other is supported by considerable empirical literature in the domain of perception and action, which has been marshaled under the common-coding theory [36]. Its core assumption is that actions are coded in terms of the perceivable effects (i.e., the distal perceptual events) they should generate. This theory also states that perception of an action activates action representations to the degree that the perceived and the represented action are similar [37]. Furthermore, these representations may be shared between individuals. Indeed, the meaning of a given object, action, or social situation may be common to several people and may activate corresponding distributed patterns of neural activation in their respective brains [38,39]. This sharing explains how we come to understand each other; that is, the isomorphism between action representations allows the individual to implicitly know the goal of others through the use of her or his own action representation system.

In neurophysiology, direct evidence for the perception/action coupling ranges from electrophysiological recordings in monkeys in which mirror neurons in the ventral premotor and posterior parietal cortices fire during both goal-directed actions and observation of the same actions performed by another individual [40], to functional neuroimaging experiments in humans that demonstrate that the neural circuit involved in action execution overlaps with that activated when actions are observed [41]. This neural network includes the premotor cortex, the parietal lobule, the supplementary motor area and the cerebellum. In addition, a number of neuroimaging studies have shown that similar brain areas are reliably activated while imagining one's own action, imagining another's action, and imitating actions performed by a model [42,43].

The perception-action mechanism accounts (at least partly) for emotion sharing, which constitutes the core mechanism for empathy [28]. This model posits that perception of emotion activates in the observer the neural mechanisms that are responsible for the generation of similar emotion. Such a system prompts the observer to resonate with the emotional state of another individual, with the observer activating the motor representations and associated autonomic and somatic responses that stem from the observed target, i.e., a sort of inverse mapping. For example, while watching someone smile, the observer activates the same facial muscles involved in producing a smile at a subthreshold level and this would create the corresponding feeling of happiness in the observer. There is evidence for such a mechanism in the recognition of emotion from facial expression. For instance, viewing facial expressions triggers expressions on one's own face, even in the absence of conscious recognition of the stimulus [44,45]. Making a facial expression generates changes in the autonomic nervous system and is associated with feeling the corresponding emotion. In a series of experiments, Levenson, Ekman and Friesen [46] instructed participants to produce facial configurations for anger, disgust, fear, happiness, sadness, and surprise while heart rate, skin conductance, finger temperature, and somatic activity were monitored. They found that such a voluntary facial activity produced significant levels of subjective experience of the associated emotions as well as specific and reliable autonomic measures. Recently an fMRI experiment confirmed and extended these findings by showing that when participants are required to observe or to imitate facial expressions of various emotions, increased neurodynamic activity is detected in the superior temporal sulcus, the anterior insula and the amygdala, as well as in areas of the premotor cortex corresponding to the facial representation [47].

The finding of paired deficits between emotion production and emotion recognition also provides strong arguments in favor of this model. A lesion study carried out with a large number of neurological patients by Adolphs and colleagues [48] found that damage within the right somatosensory related cortices (including primary and secondary somatosensory cortices, insula and anterior supramarginal gyrus) impaired patients' ability to judge other people's emotional states from viewing their face. A study of brain-damaged individuals found that recognizing emotions from prosody draws on the right fronto-parietal cortex [49]. This finding is consistent with the hypothesis that the recognition of emotion in others requires the perceiver to reconstruct images of somatic and motoric components that would normally be associated with producing and experiencing the emotion signaled in the stimulus [49].

Moreover, there are several dramatic case studies that support the idea that the same neural systems are involved both in the recognition and in the expression of specific emotion. Adolphs and colleagues [50] investigated S.M., a 30-year old patient, whose amygdala was bilaterally destructed by a metabolic disorder. Consistent with the prominent role of the amygdala in mediating certain negatively valenced emotions such as fear, S.M. was found to be impaired in both the recognition of fear from facial expressions and in the phenomenological experience of fear. Another case, N.M, who suffered from bilateral amygdala damage and left thalamic lesion was found to be impaired in recognizing fear from facial expressions and exhibited an equivalent impairment of fear recognition from body postures and emotional sounds [51]. The patient also reported reduced anger and fear in his everyday experience of emotion. There is also evidence for paired deficits for the emotion of disgust. Calder, Keane, Manes, Antoun and Young [52] described patient N.K., with left insula and putamen damage who was selectively impaired in recognizing social signals of disgust from multiple modalities (facial expressions, non-verbal sounds, and emotional prosody), and who was less disgusted than controls by disgust-provoking scenarios. Further and direct support for a specific role of the left insula in both the recognition and the experience of disgust was recently provided by an fMRI study in which participants inhaled odorants producing a strong feeling of disgust, and in another condition, watched video clips showing the facial expression of disgust. It was found that observing such facial expressions and feelings of disgust activated the same sites in the anterior insula and anterior cingulate cortex [53].

**Figure 2:**
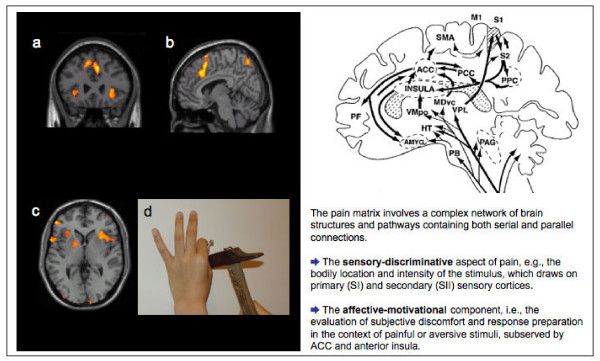
When individuals attend to the pain of others (like in b), activation is detected in the anterior ACC and SMA (a, b) and anterior insula (c). Adapted from [57]. Neurophysiological research on pain points out a distinction between the sensory-discriminative aspect of pain processing and the affective-subjective one. These two aspects are underpinned by discrete yet interacting neural networks.

The expression of pain provides a crucial signal, which can motivate caring behaviors in others. Because there is extensive behavioral and neurophysiological knowledge about the experience of pain, studying the perception of pain in others constitutes a valuable paradigm for investigating the neural mechanisms underpinning empathy. A single-neuron recording study in neurological patients has documented pain-related neurons in the anterior cingulate cortex (ACC) that respond both to actual stimulation (thermal stimuli) and also to the observation of the same stimuli delivered to another individual [54]. A first fMRI study demonstrated that the ACC, the anterior insula, cerebellum, and brainstem were activated when healthy participants experienced a painful stimulus, as well as when they observed a signal indicating that another person was receiving a similar stimulus. However, only the actual experience of pain resulted in activation in the somatosensory cortices and in dorsal ACC [55]. Similar results were also reported by Morrison and collaborators [56] in a study in which participants were scanned during a condition of feeling a moderately painful pinprick stimulus to the fingertips and another condition in which they witnessed another person's hand undergo similar stimulation. Both conditions resulted in common neuro-hemodynamic increased activity in the right dorsal ACC. This common activity in response to noxious tactile and visual stimulation was restricted to the right inferior Brodmann's area 24b. In contrast, the primary somatosensory cortex showed significant activations in response to noxious tactile, but not visual, stimuli. The different response patterns in the two areas are consistent with the ACC's role in coding the motivational-affective dimension of pain, which is associated with the preparation of behavioral responses to aversive events. These findings are supported by an fMRI study conducted by Jackson, Meltzoff and Decety [57] in which participants were shown still photographs depicting right hands and feet in painful or neutral everyday-life situations, and asked to imagine the level of pain that these situations would produce. Significant activation in regions involved in the affective aspects of pain processing, notably the dorsal ACC, the thalamus and the anterior insula was detected, but no activity in the somatosensory cortex (see Figure 2). Moreover, the level of activity within the dorsal ACC was strongly correlated with participants' mean ratings of pain attributed to the different situations.

In a follow up fMRI study, Jackson and collaborators [58], again using pictures of hands and feet in painful scenarios, instructed the participants to imagine and rate the level of pain perceived from two different perspectives (self versus other). Results indicated that both the self and the other perspectives are associated with activation in the neural network involved in the processing of the affective aspect of pain, including the dorsal ACC and the anterior insula. However, the self-perspective yielded higher pain ratings and involved the pain matrix more extensively, including the secondary somatosensory cortex, the mid-insula, and the caudal part of the anterior cingulate cortex. Adopting the perspective of the other was associated with increased activation in the right temporo-parietal junction. In addition, distinct subregions were activated within the insular cortex for the two perspectives (anterior aspect for others and more posterior for self). These neuroimaging data highlight both the similarities and self-other distinctiveness as important aspects of human empathy. The experience of pain in oneself is associated with more caudal activations (within area 24), consistent with spino-thalamic nociceptive projections, whereas the perception of pain in others is represented in more rostral (and dorsal) regions (within area 32). A similar rostro-caudal organization is observed in the insula, which is consistent with its anatomical connectivity and electrophysiological properties [59]. For instance, painful sensations are evoked in the posterior part of the insula (and not in the anterior part) by direct electrical stimulation of the insular cortex in neurological patients [60]. Altogether, these findings are in agreement with the fact that indirect pain representations (as elicited by the observation of pain in others) are qualitatively different from the actual experiences of pain.

However, studies using transcranial magnetic stimulation (TMS) reported changes in corticospinal motor representations of hand muscles in individuals observing needles penetrating hands or feet of a human model (e.g., [61]). This indicates that the observation of pain can involve sensorimotor representations. These findings are at odds with fMRI studies of empathy for pain, which did not detect any changes in the somatosensory cortex during the perception of pain in others. It is possible that the TMS method senses subtle changes in the sensorimotor cortex that exist below the significance threshold in fMRI techniques. A recent magnetoencephalographic study indicated that somatosensory oscillations are modulated by the perception of pain in others, and supports the idea that pain perception elicits subtle somatosensory activity [62]. Actually, a recent fMRI study using the same painful picture task found activation extending to the primary somatosensory areas in healthy participants [63]. It is also known that attending to a specific body part elicits somatosensory activity in the corresponding region. This has been demonstrated in a positron emission tomography study in which participants were instructed to focus their attention either on the unpleasantness or the location of the noxious stimuli delivered on the participants' hands – with the latter condition resulting in increased regional cerebral blood flow in the contralateral primary somatosensory cortex [64].

Altogether, shared representations between self and other at the cortical level have been documented for action understanding, some aspects of emotion, and pain processing. This mechanism offers a foundation for intersubjectivity because it provides a functional bridge between first-person information and third-person information [38], which allows an automatic and non-conscious connection between the self and the other. There is no specific cortical site for shared representations: their neural underpinnings are widely distributed, and the pattern of activation (and also presumably deactivation) varies according to the processing domain, the particular emotion, and the stored information. However, such a mechanism is necessary but not sufficient for empathic understanding. The awareness that others exist as separate entities is a prerequisite component of empathy.

## Self/other awareness

Individuals who are self-aware, as evidenced by being able to become the object of their own attention, experience a sense of psychological continuity over time and space [65]. It has been speculated that any organisms capable of self-recognition would have an introspective awareness of their own mental states and the ability to ascribe mental states to others [66]. A clear sense of self may have evolved to solve at least two kinds of adaptive problems: 1) the self is the repository of the social feedback one receives from others and, 2) it allows one to model and understand the internal, subjective worlds of others, making it easier to infer intentions and causes that lay behind observed behaviors, thus improving interaction efficacy [67]. Interestingly, the development of self and other mental state understanding is functionally linked to that of executive functions, i.e., the processes that serve to monitor and control thought and actions, including self-regulation, planning, cognitive flexibility, response inhibition, and resistance to interference [68]. There is increasingly clear evidence of a specific developmental link between the development of mentalizing and improved self-control at around the age of 4 [69]. The development of cognitive control is related to the maturation of the prefrontal cortex [70]. In addition, there is hard evidence that a region around the paracingulate sulcus in the medial prefrontal cortex plays a specific role in mentalizing. This region contains spindle cells, a class of large projection neurons found only in great apes and humans, which are thought to be involved in coordinating widely distributed neural activity involving emotion and cognition [71]. This region has been found to be reliably activated by mentalizing tasks of various cognitive difficulties, ranging from judging the emotion in another person's gaze, to detection of intention in simple dynamic animations, attribution of intention to cartoons characters, story comprehension, detection of social transgression, and appreciation of humor [72].

Self-awareness does not rely on a specific brain region. Rather, it arises from the interaction between processes distributed in the brain, especially the prefrontal cortex and the inferior parietal lobule. Neuropsychological research supports a preeminent role for the right frontal lobe in self-related processing. For instance, Keenan and colleagues [73] demonstrated that patients undergoing a Wada test were temporarily desensitized with regard to the recognition of their own faces when the right hemisphere was anaesthetized. This was not the case when the left hemisphere was anesthetized. Right hemisphere damage has also been found to be linked with impairments in autobiographical memory and self-evaluation. Furthermore, clinical examination has shown that personal confabulation (akin to the creation of fictitious stories about the self) appears to be associated with damage to the right frontal lobe [74].

Based on these numerous studies (and many others not reviewed here), it was argued that the right hemisphere is a key player in self-awareness and mental state attribution [75]. It is worth noting that their definition of consciousness includes awareness of one's own thoughts as well as awareness of others thoughts. Similar (but not identical) neural processing for self and other raises the question of how we distinguish between representations activated by the self and those activated by others.

Neuroscience research indicates that the right inferior parietal cortex, in conjunction with prefrontal areas and the insula, may be critical in distinguishing the self from the other and therefore in navigating shared representations. The inferior parietal cortex is a heteromodal association area, which receives input from the lateral and posterior thalamus, as well as visual, auditory, somesthetic, and limbic areas. It has reciprocal connections to the prefrontal cortex and to the temporal lobes [76]. These multiple connections confer on this region a role in the elaboration of an image of the body in space and in time [77], on which the sense of agency depends. Accumulating empirical evidence indicates that the parietal cortex plays a major role in the sense of agency in distinguishing between self-produced actions and actions generated by others [78,79] (for reviews). Interestingly, the right inferior parietal cortex at the right temporo-parietal junction is also involved when participants mentally simulate the actions from a third-person perspective in comparison with first-person perspective [80]. There are new findings suggesting that this mechanism is also at play during thinking about others. For instance, it has been demonstrated that when participants are asked to adopt another person's perspective to evaluate that person' beliefs [81], imagine his or her feelings [82] and imaging his or her pain [57,59] as compared with their own perspective, the right inferior parietal cortex is strongly involved.

**Figure 3:**
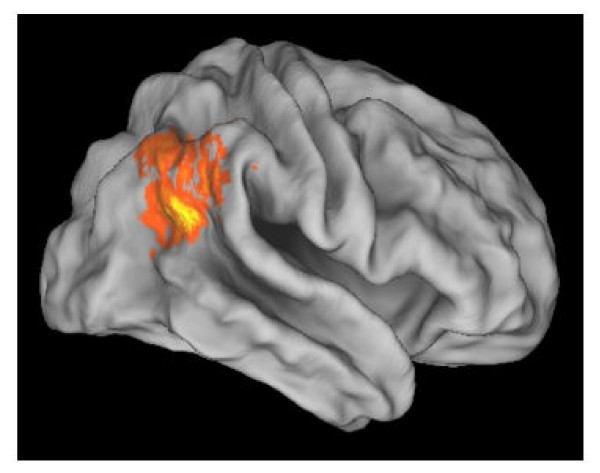
Overlap of activation between reorienting, empathy and theory of mind, projected on a partially inflated lateral view of the PALS-B12 brain atlas. The yellow to orange colors code the probability of activation, with brighter yellow indicating higher activation probability (Adapted from [83]).

Recently, Decety and Lamm [83] conducted a quantitative meta-analysis of 70 functional neuroimaging studies on agency, empathy, theory of mind, as well as on reorienting of attention. The results demonstrate a substantial overlap in brain activation between low-level processing such as reorienting of attention or the sense of agency and higher-level social-cognitive abilities such as empathy or theory of mind (see Figure 3). These results provide strong empirical support for a domain-general mechanism implemented in the right TPJ, and show that this area is also engaged in lower-level (bottom-up) computational processes associated with the sense of agency and reorienting attention to salient stimuli.

All the aforementioned evidence strongly suggests that the inferior parietal cortex, in conjunction with the prefrontal cortex and the anterior insula, plays a pivotal role in the sense of self by comparing the source of sensory signals (whether they originate from the self or from the environment). Such a function is crucial for empathy in order to maintain a minimal distinction between the self and the other and to keep track of the origin of the feelings. Self-awareness and agency are crucial for navigating the shared nature of our joint representations, and are essential properties of any autonomous agent. Indeed, social cognition relies both on similarities and differences between individuals.

## Mental flexibility and perspective taking

Empathy may be initiated by a variety of situations, for instance by seeing another person in distress or in discomfort, by imagining someone else's behavior, by reading a narrative in a fiction book or by seeing a moving TV-report. However, in these conditions, empathy requires one to adopt more or less consciously the subjective point of view of the other in relation to oneself.

Several social psychologists have suggested and documented through empirical work that our default mode to reasoning about others is biased toward self-perspective and that this constitutes a general feature of human cognition (e.g., [84,85]). Self-knowledge may serve as an anchor point for understanding others [86]. Stated in other words, people are fundamentally egocentric and have difficulty getting beyond their own perspective when anticipating what others are thinking or feeling, especially when trying to understand the states of mind of people who are perceived as being similar to themselves. Usually people are unaware of this projective tendency, which also applies to goals. This view is consistent with the shared representations mechanism. One sees others through one's own embodied cognition and uses one's own knowledge (including beliefs, opinions, attitudes, and feelings) as the primary basis for understanding others [17,38]. Self-perspective may thus be considered as the default mode of the human mind. It is a very parsimonious and advantageous mechanism for understanding and predicting the behavior of others. Yet it is far from perfect, as individual differences in people's thoughts and emotions abound. Errors in taking the perspective of others stem from the inability to suppress the self-perspective, and many costly social misunderstandings are rooted in people's failure to recognize the degree to which their construals of a situation may differ from those of others [17].

For successful social interaction, and empathic understanding in particular, an adjustment must operate on these shared representations. Whereas the projection of self-traits onto the other does not necessitate any significant store of knowledge about the other, empathic understanding requires the inclusion of other characteristics within the self. An essential aspect of empathy is to recognize the other person as like the self, while maintaining a clear separation between self and other. Hence, mental flexibility and self-regulation are important components of empathy. One needs to calibrate one's own perspective that has been activated by the interaction with the other, or even by its mere imagination. Such calibration requires the prefrontal cortex executive resources, as demonstrated by neuroimaging studies in healthy participants as well as neuropsychological observations.

Several neuroimaging studies have consistently reported that the medial prefrontal cortex is specifically involved in tasks requiring the processing of information relevant to the self, such as traits and attitudes (e.g., [87]). An fMRI study investigated the neural regions mediating self referential processing of emotional stimuli and explored how these regions are influenced by the emotional valence of the stimulus [88]. Results showed that the self-referential condition induced bilateral activation in the dorsomedial prefrontal cortex, whereas the other referential condition induced activation in lateral prefrontal areas. Activation in the right dorsomedial prefrontal cortex was specific to the self-referential condition regardless of the valence of the words. The authors of that study proposed that one specific role of the right dorsomedial prefrontal cortex is to represent states of an emotional episodic "self" and then to process emotional stimuli with a personally relevant perspective. This proposition is in line with studies showing activations within both the left and right dorsomedial prefrontal cortex during "theory of mind" tasks [89]. Because emotions generally signal issues related to the self, subjects may use emotional cues during some theory of mind tasks to differentiate self from other; this self-related emotional processing is indicated by an increase of activity in the right dorsomedial prefrontal cortex.

However, the medial prefrontal cortex is not only involved when one reflects on oneself, but also when individuals intentionally adopt the subjective perspective of others. A series of three neuroimaging studies with healthy volunteers investigated the neural underpinning of perspective taking in three different modalities (i.e., motoric, conceptual, and emotional) of self-other representations. In a first study, participants were scanned while they were asked to either imagine themselves performing a variety of everyday actions (e.g., winding a watch), or to imagine another individual performing similar actions [80]. Both conditions were associated with common activation in the supplementary motor area (SMA), premotor cortex, and the occipito-temporal region. This neural network corresponds to the shared motor representations between the self and the other. Taking the perspective of the other to simulate his or her behavior resulted in selective activation of the frontopolar cortex and right inferior parietal lobule. In a second study, medical students were shown a series of affirmative health-related sentences (e.g., taking antibiotic drugs causes general fatigue) and were asked to judge their truthfulness either according to their own perspective (i.e., as experts in medical knowledge) or according to the perspective of a layperson [81]. The set of activated regions recruited when the participants put themselves in the shoes of a lay-person included the medial prefrontal cortex, the frontopolar cortex and the right inferior parietal lobule. In a third study, the participants were presented with short written sentences that depicted real-life situations (e.g., someone opens the toilet door that you have forgotten to lock), which are likely to induce social emotions (e.g., shame, guilt, pride), or other situations that were emotionally neutral [82]. In one condition, they were asked to imagine how they would feel if they were experiencing these situations. And in another condition, they were asked to imagine how their mothers would feel in those situations. Reaction times were statistically greater when the participants imagined emotional-laden situations as compared with neutral ones, both from their own perspective and from the perspective of their mothers. Neurodynamic changes were detected in the frontopolar cortex, the ventromedial prefrontal cortex, the medial prefrontal cortex, and the right inferior parietal lobule when the participants adopted the perspective of their mothers, regardless of the affective content of the situations depicted. Cortical regions that are involved in emotional processing, including the amygdala and the temporal poles, were found activated in the conditions that integrated emotional-laden situations. Consistent findings were reported from a functional MRI study in which participants were asked to make food preference judgments about themselves or about someone else (a person whom they knew fairly well). Self-judgments were associated with increases in the medial prefrontal cortex, the anterior insula, and secondary somatosensory areas. Judgments of the other resulted in activation of the medial prefrontal cortex, the frontopolar cortex, and the posterior cingulate [90].

A recent functional MRI study used a factorial design to examine the neural correlates of self-reflection and perspective taking [91]. Participants were asked to judge the extent to which trait adjectives described their own personality (e.g., "Are you sociable?") or the personality of a close friend (e.g., "Is Caroline sociable?") and were also asked to put themselves in the place of their friend (i.e., to take a third-person perspective) and estimate how this person would judge the adjectives, with the target of the judgments again being either the self (e.g., "According to Caroline, are you sociable?") or the other person (e.g., "According to Caroline, is she sociable?"). The results showed that self-referential processing (i.e., judgments targeting the self vs. the other person) was associated with activation in the ventral and dorsal anterior MPFC, whereas perspective taking (i.e., adopting the other person's perspective, rather than one's own, when making judgments) resulted in activation in the posterior dorsal MPFC; the interaction between the two dimensions yielded activation in the left dorsal MPFC. Findings from this study indicate that self-referential processing and perspective-taking recruit distinct regions of the MPFC and suggest that the left dorsal MPFC may be involved in decoupling one's own from other people's perspectives on the self.

One of the most striking findings of the studies that investigated self- versus other's perspective is the systematic involvement of the frontopolar cortex, medial prefrontal cortex, posterior cingulate, and right temporo-parietal junction when the participants adopt the perspective of another person (a circuit also associated with theory of mind tasks). Converging evidence from clinical neuropsychology and neuroscience points to the frontopolar cortex as being chiefly involved in inhibitory or regulating processing. Frontal damage may result in impaired perspective-taking ability and a lack of cognitive flexibility [92]. Anderson and colleagues [93] reported the cases of two patients with early damage to the anterior prefrontal cortex (encompassing the frontopolar cortex) who, when tested on moral dilemmas, exhibited an excessively egocentric perspective. A major study of patients with limited focal lesions to the frontal lobes, who were tested for visual perspective taking and detecting deception, revealed a dissociation of performance within the frontal lobes [94]. Right frontal lobe lesions were associated with impaired visual perspective taking, whereas medial frontal lesions, particularly right ventral, were associated with impaired detection of deception.

Overall, these findings support the hypothesis that an inhibitory component is required to regulate and tone down the self-perspective tendency and allow the cognitive and affective flexibility necessary to the evaluation of the other's perspective. Such a view is compatible with the role of the prefrontal cortex in top-down control of behavior [95]. An alternative interpretation of the role of the frontopolar cortex in adopting the perspective of another individual is based on the distinction between different psychological operations mediated by distinct subregions of the prefrontal cortex. There is evidence that the frontopolar cortex is involved in the process of evaluating self-generated responses, and is recruited when the task requires monitoring and manipulation of information that has been internally represented [96]. Adopting the subjective perspective of another individual to understand her feelings is a self-generated process that operates on internally represented information fed by the internal activation of shared representations.

## Emotion regulation

The capacity to regulate one's own emotions has a clear adaptive function for social interaction, both for the individual and the species. It has been demonstrated that individuals who can regulate their emotions are more likely to experience empathy, and also to act in morally desirable ways with others [97]. Emotion regulation refers to the processes by which individuals influence which emotions they have, when they have them, and how they experience and express these emotions [98]. It also applies to the modulation of the behavioral and the physiological dimensions of emotion.

It is likely that the emotional state and affective consequences generated in the self from the perception or imagination of the other's affective state requires some regulation and control for the experience of empathy. Indeed, without such control, the mere activation of the perception-action mechanism, including the associated autonomic and somatic responses, could lead to emotional contagion or emotional distress. Such regulation is also important in modulating one's own vicarious emotion so that it is not experienced as aversive. Previous research indicates that emotion regulation is positively related to feelings of concern for the other person [97,99]. In contrast, individuals who experience their emotions intensely, especially negative emotions, are prone to personal distress, i.e., an aversive emotional reaction such as anxiety or discomfort based on the recognition of another's emotional state or condition [100]. Chronic incapacity to suppress negative emotion may be a key factor in anxiety, and aggressive and violent behavior [101].

A circuit that includes several interconnected regions of the prefrontal cortex, the amygdala, hippocampus, ACC, insular cortex, and ventral striatum has been acknowledged to be implicated in various aspects of emotion regulation [102]. In neurology, the term "self-regulatory disorder" has been coined for the syndrome exhibited by patients with ventromedial prefrontal cortex damage (particularly on the right). This syndrome is defined as the inability to regulate behavior according to internal goals and constraints [103]. It arises from the inability to hold a mental representation of the self on-line and to use this self-related information to inhibit inappropriate responses. Interestingly, the orbitofrontal, ventromedial, and dorsolateral cortices have been reported in the neurological literature to be involved in empathy. Notably, damage to the orbitofrontal is associated with a wide range of social emotional deficits, including impaired social judgment and disinhibited behavior. For instance, Stone, Baron-Cohen and Knight [104] found that patients with bilateral lesions of the orbitofrontal cortex are impaired in the "faux pas" task. This task requires both an understanding of false or mistaken belief and an appreciation of the emotional impact of a statement on the listener. A study conducted by Stuss and colleagues [94] extended this finding by showing that only lesions in the right orbitofrontal produce such a deficit. In addition, a number of clinical studies reported a relationship between the deficit in empathy and poor performance of cognitive flexibility tasks among patients with lesions in the dorsolateral regions, whereas those with orbitofrontal cortex lesions were more impaired in empathy but not in cognitive flexibility [105,106]. The ventromedial prefrontal cortex with its reciprocal connections with brain regions involved in emotional processing (amygdala), memory (hippocampus), and executive functions (dorsolateral prefrontal cortex) plays also a major role in emotion regulation. Damasio's [107] somatic markers hypothesis, which posits that memories of somatic states that are associated with particular experiences or outcomes are stored in the ventromedial prefrontal cortex, is directly relevant in the process of affective regulation. Recent work by Shamay-Tsoory and colleagues [108] supports this hypothesis. They tested patients with lesions of the ventromedial prefrontal cortex or dorsolateral prefrontal cortex with three theory-of-mind tasks (second-order beliefs and faux pas) that differed in the level of emotional processing involved. They found that patients with ventromedial lesions were most impaired in the faux pas task but presented normal performance in the second-order belief tasks. The authors further argued that in order to detect faux pas, one is required not only to understand the knowledge of the other but also to have empathic understanding of other's feelings. Finally, the ACC is part of a circuit involved in a form of attention that serves to regulate both cognitive and emotional processing [109]. Its lesion produces a host of symptoms, which include apathy, inattention, dysregulation of autonomic functions, and emotional instability.

Neuroimaging research has recently begun to investigate neural mechanisms involved in affective reappraisal, a cognitive strategy used to regulate emotion. For instance, an fMRI experiment on emotion reappraisal has detected co-activation of the lateral prefrontal and medial prefrontal cortices and decreased activity in the medial orbitofrontal cortex and the amygdala [110]. Another study identified a circuit composed of the right orbitofrontal, right dorsolateral prefrontal cortex and anterior cingulate for voluntary suppression of sadness [111]. One recent functional MRI study investigated whether observation of distress in others leads to empathic concern and altruistic motivation or to personal distress and egoistic motivation [112]. In this experiment behavioral measures and event-related functional MRI were used to explore the effect of perspective taking and emotion regulation on empathy processing while participants watched video-clips of patients expressing pain resulting from medical treatment. Video-clips were presented either with the instruction to imagine the feelings of the patient ("imagine other") or to imagine oneself to be in the patient's situation ("imagine self"). Need for emotion regulation was manipulated by providing information that the medical treatment had or had not be successful. Behavioral measures clearly demonstrated that imagery and reappraisal instructions were effective. Neuroimaging data showed consistent activity in the insular cortex and anterior medial cingulate cortex (aMCC). Graded responses related to the imagery instructions were observed in dorsal insula, aMCC, and left and right parietal cortex. Emotion regulation resulted in hemodynamic changes in anterior paracingulate cortex, subgenual ACC, orbitofrontal, and right temporal cortex. These findings support the view that the response to the pain of others can be modulated by cognitive and motivational processes. These processes influence whether observing a conspecific in need of help will result in empathic concern, an important prerequisite for helping behavior.

Overall, the capacity to regulate emotions is an important aspect of our ability to interact appropriately with other people. The prefrontal cortex is highly differentiated in terms of cell structures and patterns of interconnectivity with other cortical subsystems. In line with this fact, neuroimaging studies suggest that specific systems interact in generating emotion regulation. Social neuroscience is beginning to shed light on the physiological and neural mechanisms subserving the various emotion regulation strategies that allow us to better understand our conspecifics.

## Empathy relies on intersubjective awareness

The way our nervous system is organized and tailored by evolution provides the basic biological mechanism for resonating with the behaviors of others. This mechanism driven by the common coding between perception and action provides the default mode to implicitly relate to others and may be responsible for the projective tendency to ascribe one's own characteristics and self-traits to others [38,113]. However, this tendency needs to be regulated (or calibrated) for appropriate social interaction [17]. This requires additional computational mechanisms, including monitoring and manipulation of internal information generated by the activation of the shared representations between the self and the other. In addition, there are limits to the extent to which the experiences are isomorphic, as demonstrated by the non-overlapping neural areas.

One of the core components of empathy relies on the unconscious neural/mental simulation of the emotional state of others. This idea is far from new (e.g., [107,114,115]). For instance, Ax [116] in 1964 had suggested that empathy might be thought of as an autonomic nervous system state, which tends to simulate that of another person. This idea fits neatly with the notion of embodiment, which refers both to actual bodily states and to simulations of experience in the brain's modality-specific systems for perception, action, and the introspective systems that underlie conscious experiences of emotion, motivation and cognitive operations [117]. However, this simulation is not exclusively under automatic management and, at least in humans, falls under conscious control. This makes empathy, as described here, an intentional capacity. Without self-awareness and emotion regulation processing, there may be no true empathy. The automatic activation of shared representations would instead be associated with anxiety and discomfort and would lead to responses oriented to the self (e.g., emotional distress). Such a formulation is also consistent with the observation that prosocial behaviors, which stem from empathy, emerge during child development in parallel with self-conscious emotions [118]. These emotions require self-evaluation and comparison with others, as well as some form of emotion regulation. Forming an explicit representation of another person's feeling, as an intentional agent, thus necessitates additional computational mechanisms beyond the shared representation level. This requires that second-order representations of the other are available to consciousness (a decoupling mechanism between first-person information and second-person information) [119].

Thus human empathy cannot be described only as a simple resonance of affect between the self and other. Indeed, empathy is both about sharing and understanding the emotional state of others in relation to oneself. The capacity for two people to resonate with each other emotionally, prior to any cognitive understanding, is the basis for developing shared emotional meanings, but is not sufficient for empathy. Such an understanding goes beyond this reflex-like response. It involves an explicit representation of the subjectivity of the other and a minimal self-other distinction. Recent neuroimaging investigations of the perception of pain in others support such a view (e.g., [55,57,58,112]). Indeed, all these studies have shown that part of the neural network (including the anterior cingulate cortex and the anterior insula) mediating self-experienced pain is shared when empathizing or observing the pain in others, and also that non-overlapping aspects within these regions are specifically activated for the self or the other. This supports the idea that personal and vicarious experiences at some level differ physiologically [120] and result in qualitatively distinct responses. Finally, empathy also necessitates emotion regulation in which the ventral prefrontal cortex, with its strong connections with the limbic system, dorsolateral, and medial prefrontal areas, plays an important role.

We believe that a greater understanding of the underlying computational processes and their neural underpinnings can contribute to a better characterization of empathy disorders in psychopathology.

## Empathy disorders and psychopathology

The empirical evidence reviewed here illuminates the neurobiological underpinnings of the different components of empathy and strongly suggests the view that lesion to different cortical and sub-cortical structures or circuits can lead to an alteration of empathy or even a lack of empathic ability. However qualitative differences exist in the nature of underlying deficits, and this supports our assertion that empathy entails a number of distinct components mediated by isolable neural systems. It is possible that psychopathological disorders such as antisocial personality disorders, schizophrenia or autism, in which social breakdown are predominant for various reasons, will benefit from this integrative model. The clinical imperative is to understand the factors that lead to these conditions, uncover what neural mechanisms may underlie these deficits, and hence treat them.

Many psychiatric disorders are associated with deficits or even lack of empathy. Here, we will discuss a limited number of these disorders including psychopathy/antisocial personality disorders, borderline and narcissistic personality disorders, autistic spectrum disorders, and alexithymia.

### Psychopathy and antisocial personality disorder (ASP)

Psychopathy is a disorder that encapsulates the essence of a lack of empathy. The classification of psychopathy, introduced by Hare [121], involves both affective-interpersonal (e.g., lack of empathy and guilt) and behavioral components (e.g., criminal activity and poor behavioral control).

Empathy deficit in antisocial personality disorder has been suggested to come from a reduced ability to feel other people's emotional state, and more so for sadness and fear [122]. This deficit has been ascribed to a dysfunction in the amygdala of developmental origin. This view is compatible with the fact that individuals with this disorder have generally intact executive functions and can successfully complete Theory of Mind tasks [121]. There is also evidence to suggest that psychopathic patients do well on the 'Reading the Mind in the Eyes' task by simply asking them to focus on the eyes of other target people [123]. Therefore, their lack of empathy could be related to disrupted affective processing rather than an inability, for instance, to adopt the perspective of others. In fact, people with antisocial personality disorders are probably good at perceiving others' intentions, while disregarding the emotional content, however, and thus may take advantage of it. This is precisely what the research of Mealey [124] suggests. The psychopath cannot simulate emotions he cannot experience, and must rely exclusively on cognitive inputs to his theory of mind mechanism.

An interesting single case of acquired sociopathy has been reported by Blair and Cipolotti [125]. The authors investigated an individual, J.S., with orbitofrontal cortex and left amygdala damage with an impressive battery of measures, including skin conductance response (SRC), tests of executive functions, emotion recognition and social cognition tasks. While J.S. showed executive impairments but no reversal learning impairment, he was significantly impaired on most of the social cognition tasks. Notably, he was both impaired in the recognition of emotional expressions (happiness, anger, disgust and sadness) and in the attribution of emotional states to others (fear, anger and embarrassment). His ability to attribute mental states to others was preserved. His SRC responses to negative emotional expressions were reduced. Blair and Cipolotti argued that the distinctive features of the acquired sociopathy of J.S. were due to impairment of a system which responds to angry expressions/expectations of others' anger, and that this system is particularly involved in the suppression of socially aberrant behavior.

Antisocial personalities are often reported to perform poorly on neuropsychological tests of executive functioning (e.g., [126]). Executive functions are considered necessary for socially appropriate conduct, and in our framework, they contribute to empathy through self-regulation. In line with this view, a meta-analysis of thirty-nine studies (yielding a total of 4589 participants) helped to clarify the relation between antisocial behavior and executive functions [127]. The results of this meta-analysis indicate that there is a robust and statistically significant relation between executive functions and antisocial behavior. The authors were unable to subdivide executive function measure in terms of their associations with different brain regions (e.g., dorsolateral, orbitofrontal) because of the lack of knowledge concerning the neuroanatomical substrates of most executive functions tasks. Interestingly, Blair [122] proposed that people with antisocial personalities have a disruption of a violence inhibition mechanism that is normally triggered by distress cues of others, and this aspect belongs to executive functioning.

Clinical and forensic research usually distinguish "affective" or "reactive" aggression, which is a response to physical or verbal aggression initiated by others with violence that is relatively uncontrolled and emotionally charged, from a "predatory" or "instrumental" cold-blooded aggression, which is a controlled, purposeful aggression lacking in emotion that is used to achieve a desired goal [128]. Our model of empathy predicts that the former type of personality would lack executive control (particularly self-control) and emotion regulation, whereas the latter personality would have some dysfunctions in sharing feelings with others. Interestingly, measurements of glucose metabolism in two groups of affective and predatory murderers have shown that the first group has lower prefrontal activity, and the second group has similar prefrontal activity as compared to controls, but lower activity at the subcortical level including the amygdala [129].

### Borderline and narcissistic personality disorder (BPD/NPD)

Borderline personality disorder (BPD) is characterized by emotional dysregulation, "splitting" (or black and white) thinking, and fluctuating and chaotic interpersonal relationships, including pervasive instability in mood, interpersonal relationships, identity, and behavior, as well as a disturbance in the individual's sense of self. They often have severe deficiencies in impulse control, which results in self-destructive behaviors, burst aggression to others. Abnormalities of brain function have been assumed in the areas associated with cognition, affect, emotion regulation, and impulsivity, particularly in the prefrontal cortex and the temporal lobe, including the amygdala and/or hippocampus. In accord with this notion, patients with BPD showed enhanced amygdala activation in response to standardized emotionally aversive pictures [130]. Donegan et al [131] reported that BPD patients showed significantly greater left amygdala activation to the facial expressions of emotion compared with healthy subjects, and in post-scan debriefing some patients had difficulty disambiguating neutral faces or found them threatening. Minzenberg and colleagues [132] also confirmed BPD group exhibiting significantly greater activation in the right amygdala to fear minus neutral facial expressions, and a significantly larger magnitude of deactivation on (relative to healthy control) in the bilateral rostral/subgenual anterior cingulate cortex (ACC) to fear and in the left ACC to fear minus neutral; and they concluded that BPD patients exhibit changes in fronto-limbic activity in the processing of fear stimuli, with exaggerated bilateral amygdala response and impaired emotion-modulation of ACC activity. A FDG-PET study demonstrated a tight coupling of glucose metabolic activity in resting condition between right OFC and ventral amygdala in healthy subjects with dorso-ventral differences in amygdala circuitry, but not present in BPD group, without any significant differences in amygdala volumes or metabolism between BPD patients and controls.

All these studies described above suggest that BPD includes 'hyper-sensitivity' in the limbic system primarily including the amygdala and a hypofunction of the prefrontal cortices. In the context of empathy, both emotional and cognitive function and interaction between two components may be altered in patients with BPD.

From a clinical point of view, Kernberg [133] proposed psychopathological diagnostic criteria of borderline personality organization: impaired ego integration with "primitive defenses" like 'splitting', in which a person or thing is seen as alternative and fluctuating 'all good' or 'all bad', problems with object constancy and continuity in people and things in their lives, and 'projection' of unpleasant characteristics in the self onto others and 'projective identification', a process where the borderline tries to elicit in others the feelings he/she is having. These notions are in line with their self-oriented immature level of empathy (e.g., personal distress or posing their mental state on the other), that is, over-affective and exaggerated resonance with other's mental state with lowered top-down modulation by correct meta-cognitive process.

People with narcissistic personality disorder (NPD) also have difficulty recognizing the needs and feelings of others, and are dismissive, contemptuous and impatient when others share or discuss their concerns or problems. They are also oblivious to the hurtfulness of their behavior or remarks, show an emotional coldness and a lack of reciprocal interest, exhibit envy (especially when others are accorded recognition), have an arrogant, disdainful and patronizing attitude, and are quick to blame and criticize others when their needs and expectations are not met. Although there is scarcely neuroscientific evidence in NPD (c.f., [134]), underpinnings of the lack of empathy in NPD seem common with that in BPD, although it is supposed that BPD contains dysregulation in more affective aspects than NPD from empirical clinical observation. It was shown that abusive parents are more likely to lack parental warmth, compassion and concern and experience difficulty in perspective taking, and at the same time, have less self-confidence, a greater lack of impulse control and are more narcissistic [135]. It is assumed that patients with NPD might have reduced affective neural component of empathy. Further evidences are needed to validate this hypothesis.

### Autism and Autistic spectrum disorders (ASD)

Autistic spectrum disorders (ASD) is a severe developmental disorder where there is marked neuro-cognitive impairment. Children with ASD display a broad range of social communication deficits, and most scholars agree that a lack of empathy (taken in a very broad sense) prominently figures amongst them. The underlying cause of the empathy deficit is, however, more controversial. For instance, Baron-Cohen and colleagues [136] put forward the hypothesis that the social impairment in autism arises from a failure of a mentalizing mechanism (a theory-of-mind module). Other authors believe that children with autism have a hard time feeling and expressing emotion, and that this basic deficit prevents them from engaging in social interactions [137]. Others still, like Russell [68], argue that deficits in executive functions are the major cause for the social/communicative disorders observed in autism. Rogers and Pennington [138] suggested a cascade model of autism in which the lack of certain aspects of interpersonal development at every previous stage disrupts certain developments in the following stage. These authors view early imitation skills, emotion sharing, and theory of mind as increasingly complex expressions of the ability to form and coordinate certain representations of self and other. These representations are then used to guide the planning and execution of one's own behavior. Finally, Dawson [139] proposed that autism involves impairment in attentional functioning for social stimuli (e.g., facial expressions, speech, gestures). She hypothesized that, because social stimuli are complex, variable, and unpredictable, children with autism have difficulty processing and representing them and, therefore, their attention is not naturally drawn to such stimuli [140]. These different views (imitation/emotions sharing *vs*. executive functions) may not be as incompatible as it seems. Indeed, it remains possible that empathy deficits in autism are related to disruption of either emotion sharing or mental flexibility/self regulation components, or even both.

Several studies have examined behavioral and autonomic responses of children with autism who look at adults depicting facial emotional expressions (see [141] for a critical review). While most studies report that children with autism look less frequently at the adult faces than control subjects in empathy-eliciting situations, the remaining findings are equivocal. For instance, one study did not find any change of heartbeat rate during the observation of someone in distress [142]. Another study has shown that the autonomic responses of these children change according to the distress of the target, if the emotions displayed are not ambiguous and if they are presented under conditions with reduced distraction [143]. Moreover, and contrary to what is often claimed, children with autism can make moral/conventional distinction [144]. It is likely that these children present a difficulty in taking the perspective of others, which requires executive resources, but they seem to have the physiological substrate to display affective sharing abilities. Altogether, both impairment in executive functions and emotion sharing may account for the empathy deficit in autism.

There is also evidence of deficits in the perception-action coupling, corresponding to basic level of empathy, like mimicry. One study has shown that when compared with developmentally delayed children, 20-month-old infants with autism were found to be specifically impaired on empathy task, joint attention and imitation [145]. Imitation deficits have been proposed to explain the difficultly of autistic children in establishing social relationships and identifying with others [138]. For instance, a study by Hobson & Lee [146] demonstrated that autistic children can imitate the goal of actions displayed by an experimenter but failed to imitate the affective style with which the actions were carried out. This suggests that these children cannot readily identify with the experimenter's perspective entirely. It has also been demonstrated that long before children with autism show theory of mind deficits, they exhibit deficits in joint attention and attention monitoring [147]. A study examined 30 to 70 month-old autistic and healthy children's social behavior, affect, and use of gaze during naturalistic interactions with their mothers [148]. Both autistic and typically developing children responded with smiles more frequently to social events than to nonsocial events. However, when autistic children's responses to the mother's smiles were examined, the authors found that they never smiled in response to the mother's smile. In other words, they do not exhibit the biologically based ability to automatically resonate with others which consists of very basic level of empathy, indicating their perception-action coupling deficits in a behavior that is considered as a manifestation of emotion contagion, like facial mimicry. Another recent study found that individuals with autism do not show spontaneous mimicry, but they perform voluntary mimicry well [35]. Such a core deficit in involuntary motor resonance may be the seed for their profound impairment in basic emotional connectedness.

Burgeoning research efforts suggest that a deficient mirror neuron system may contribute to motor and social problems experienced in individuals with ASD. Indeed, brain areas associated with the mirror-neuron system and imitation have all been observed as aberrant in terms of structure and function in individuals with ASD. Indeed, recent research with humans using transcranial magnetic stimulation (TMS) demonstrates changes in the amplitude of the motor-evoked potentials during action observation, a clear demonstration of the motor resonance mechanism (e.g., [149]). To build on this finding, TMS was applied over the motor cortex of adults with ASD and matched healthy controls. Compared to the controls, adults with ASD showed significantly less M1 activation during the observation of transitive, meaningless finger movements [150]. In contrast, observation of the finger movements in control subjects yielded enhanced M1 activity in areas delivering signals to the muscles concerned with the observed action. The weaker M1 modulation in individuals with ASD suggests that the less mirror neuron activation in the motor cortex may be partly responsible for the deficits in social cognition, specifically abnormal self-other representations, diminished reciprocal social capacities, and hindered development of empathy. In attempt to examine a potential link between mirror neuron dysfunction and developmental delay of social cognitive skills, one fMRI study found a lack of activation in the inferior frontal gyrus (a key mirror neuron area) in children with ASD as compared to controls during the observation and imitation of basic facial emotion expression [151]. These recent findings, which need to be replicated, seem to suggest that dysfunction in the mirror neuron system may hamper the normal development of self-other connectedness, creating a cascade of deficient processes that lead to social deficits, including some aspects of empathy.

It should be noted that, to date, it is not clear what aspect of empathy is dysfunctional in ASD, and there is not enough empirical research that has addressed this question. However, as noted above, there are growing evidences suggesting that disruption of both the motor/affective and meta-cognitive self-regulational aspects of empathy. Individuals with autism appear to have deficiencies over broader aspects of empathy, although this notion needs to be clarified in the future.

### Alexithymia

Alexithymia refers to deficiencies in understanding, processing, or describing emotions in the self [152]. Since awareness of emotional states in the self is a prerequisite to recognizing such states in others, alexithymia should involve impairment in empathy. Although alexithymia is not a diagnostic disorder, it is a personal trait that is prevalent in broad psychiatric and psychosomatic spectrums, which are characterized by deficits in empathy, such as autistic spectrum disorder [153-155], schizophrenia [156-161], borderline [162], narcissistic [163] and psychopathic personality disorders [164]. Guttman et al. [162] also showed that alexithymia scale is correlated with empathy scale using borderline personality population. Moriguchi and colleagues [165] showed that alexithymic people showed low theory of mind ability when they mentalize the moving triangle animation and, using fMRI, low neural activity in medial prefrontal cortex, which is mostly associated with perspective taking scale. Moriguchi and colleagues [63] further used fMRI to compare neural response in a group of people with alexithymia with a group of healthy controls to the visual perception of pictures depicting human hands and feet in painful situations. The alexithymia group showed less cerebral activation in the left dorsolateral prefrontal cortex, the dorsal pons, the cerebellum, and the left caudal anterior cingulate cortex within the pain matrix. The alexithymia group showed rather greater activation in the areas related to affective processing such as the right anterior and posterior insula and inferior frontal gyrus. Furthermore, alexithymic participants scored lower on the pain ratings and on the scores related to cognitive mature empathy. Another fMRI study [166] also showed that a population with alexithymia had lower neural activity in medial prefrontal cortex in response to affective pictures, but there is no difference of activation in the brain areas associated with bottom up information processing such as limbic structures (i.e., the amygdala, the hippocampal formation, and the hypothalamus), which play a central role in emotional response to simple perceptual and associative aspects of the stimuli. In sum, alexithymia has certain deficits in empathy, particularly in the aspects of mental flexibility to adopt the subjective perspective of the other and executive and regulatory processes that modulate the subjective feelings associated with emotion. These facts also support the importance of self-awareness in empathy, and these cognitive impairments underlie and constitute core psychopathology related to the social and interpersonal difficulties in various psychiatric disorders listed above.

Taken together, the available empirical evidence from the various forms of empathy disorders in psychiatry population fits neatly with a multiple-component model. It further reveals that there are important differences in the cognitive and neural systems involved in the cognitive and affective architecture of empathy and its social behavioral outcomes. We believe that such an approach is an invaluable research tool with respect to the understanding, and ultimately the therapy/treatment of this disorder.

## Conclusion

Empathy is a fundamental ability for social interaction and moral reasoning. It refers to an emotional response that is produced by the emotional state of another individual without losing sight of whose feelings belong to whom. This response is contingent on cognitive, as well as emotional, factors and involves parallel and distributed processing in a number of dissociable computational mechanisms. Like many complex experiences, empathy emerges from the flow and integration of information between specific brain circuits. Current trends in empathy theory suggest that it involves partly dissociable components, including shared neural affective representations, self-awareness, mental flexibility, and emotion regulation. These basic macro-components of empathy are mediated by specific and interacting neural systems.

These macro-components may comprise more elementary components that future social neuroscience studies in combination with clinical research will elucidate. Moreover, because this model assumes that empathy relies on dissociable information processing components, it predicts a variety of structural or functional dysfunctions depending on which aspect is disrupted. Indeed, there are various forms of empathy dysfunctions in psychopathology such as antisocial personality disorders, psychopathy, narcissistic personality disorders and autism, which seem to reflect selective impairment of one or several components of the neurocognitive architecture of empathy.

The lack of empathy leads to profound disturbance and dysfunction in social interaction, and hence is important to study in the domain of psychopathology. Future clinical investigations of empathy disorders can only be informative if behavioral, dispositional and biological factors are combined. Multiple levels of analysis are fundamental when addressing such a complex aspect of psychology. Too often, the assessment of empathy in both healthy and psychiatric populations relies on self-report measures that alone are not valid. Further, empathy may be a prerequisite for altruism, but the relation between empathy and prosocial motivation is far from being simple and direct. Both biology and experience contribute to empathy and sympathy in complex interactive, bidirectional ways.

Finally, one of the challenges for a social neuroscience approach to empathy and its disorders is the difficulty of taking into account situational variables. To provide interpretable data, neuroscience experiments require intra-individual comparisons and repeated-measures designs. To be financially feasible, they require small samples. These conditions limit opportunities to study the effects of potentially important situational variables. This is but one example of the perennial challenge objective science faces in the attempt to understand human subjectivity in all its richness and complexity [167].

## Competing interests

The author(s) declare that they have no competing interests.

## Authors' contributions

JD and YM participated in the writing of the manuscript. Both authors read and approved the final manuscript.

**Table 1:** Clearing up conceptual issues. Despite the abundance of definitions of empathy, it is possible and recommended to differentiate emotional contagion, empathy, sympathy and personal distress

-	Emotional contagion is tendency to automatically mimic and synchronize facial expressions, vocalizations, postures, and movements with those of another individual.
-	Empathy is an emotional response that stems from another's state and that is congruent with the other's emotional state. It involves at least a minimal distinction between self and other. Empathy is not a separate emotion by itself, but a kind of induction process by which emotions, both positive and negative, are shared, and which increase the chances of similar behaviors in the observer.
-	Personal distress is an aversive state (e.g., anxiety, worry) that has not to be congruent with the other's state, and that leads to a self-oriented, egoistic reaction.
-	Sympathy (or empathic concern) refers to feelings of sorrow, or sorry for another. It is often the consequence of empathy, although it is possible that sympathy results from cognitive perspective taking. Sympathy is believed to involve an other-oriented, altruistic motivation.
-	Emotion can be considered a process that facilitates appropriate physiological responses to aid the survival of the organism.
